# The Final Act: An Ethical Analysis of Pia Dijkstra’s Euthanasia for a Completed Life

**DOI:** 10.1007/s11673-020-10084-x

**Published:** 2021-01-15

**Authors:** T. J. Holzman

**Affiliations:** King’s College London, Strand, WC2R 2LS, London, UK

**Keywords:** Bioethics, End-of-life, Euthanasia, Completed life, Autonomy, Dying with dignity, Existential suffering

## Abstract

Amongst other countries, the Netherlands currently allows euthanasia, provided the physician performing the procedure adheres to a strict set of requirements. In 2016, Second Chamber member Pia Dijkstra submitted a law proposal which would also allow euthanasia without the reason necessarily having any medical foundation; euthanasia on the basis of a completed life. The debate on this topic has been ongoing for over two decades, but this law proposal has made the discussion much more immediate and concrete. This paper considers the moral permissibility of Pia Dijkstra’s law proposal, focusing on the ethics of the implementation Dijkstra describes in her proposal. I argue that, at present, Dijkstra’s law proposal is unsuitable for implementation, due to a number of as of yet unaddressed problems, including the possible development of an ageist stigma and undue pressure on the profession of end-of-life coordinator. Perhaps adequate responses can be conceived to address these issues. However, the existence of a radically different, yet currently equally unacceptable position regarding the implementation of euthanasia for a completed life as proposed by fellow party member Paul Schnabel suggests it may be difficult to formulate an ethically acceptable implementation for this, in principle, ethically acceptable concept.

## Introduction

Currently euthanasia in the Netherlands is not legal, it is a punishable offence. However, the physician may be pardoned from prosecution, if he adheres to six conditions. The Dutch term for these conditions can be translated as carefulness demands (zorgvuldigheidseisen) and are as follows:

1. The physician must be convinced that the patient’s request is well thought through and voluntary;
2. The physician must be convinced of the patient’s hopeless and unbearable suffering;
3. The physician must have informed the patient fully of their medical situation, both current and future prospects;
4. The physician and the patient must come to the joint agreement there is no other reasonable solution to the situation;
5. The physician must have consulted at least one other independent physician; and
6. The euthanasia must be performed in a medically careful way (Overheid 2001; Cohen-Almagor 2004).

The aims of these demands pertain mainly to the autonomy and agency of the patient, the hopelessness of the situation, and the procedure being medically safe (Overheid 2001; Kimsma and van Leeuwen 2001; Battin and Quill 2004).

In 2016, Pia Dijkstra, member of the Second Chamber, the Dutch equivalent to the House of Commons, submitted a law proposal to allow euthanasia on the basis of a completed life in addition to the existing euthanasia law (D66 2016; Voltooid Leven 2016). This means that the request for euthanasia need not necessarily have a medical foundation. This is to say, rather than unbearable and relentless suffering as a result of a physical or mental disease with a diagnosable cause, the individual simply experiences their life as complete and therefore does not wish to continue living (Wet Toetsing Levenseindebegeleiding van Ouderen op Verzoek 2016; NVVE 2015). The potential lack of a medical foundation of the death wish is crucial to the concept of a completed life. Dijkstra has publicly stressed that her proposal aims to offer respect and agency to autonomy and the freedom of elderly people to choose when and how they wish to die (NOS 2017). The proposal has sparked a nationwide controversy, featuring concern for matters such as safety, lack of clarity, potential misuse, autonomy, and freedom of choice (Van Steenbergen, 2017a, 2018; van Houten 2018; van Ast 2017; Sanders 2017; Blanken 2018; Kieskamp 2017; McLachlan 2011).

Dijkstra’s proposal was met with adversity by her fellow party member, Paul Schnabel, member of the First Chamber, or Dutch equivalent to House of Lords. Schnabel and his advice committee advised against proposing a new law. Their view is that the Dutch euthanasia law was written in such a way that there is room for expansion and development. Initially it was exclusively intended for terminal patients, and now it has expanded to also include chronic patients and patients suffering from an accumulation of age-related complaints. They argue that it will most likely also expand to include this unwillingness to continue living despite the lack of any medical condition as a cause. A law proposal which aims this option specifically at the elderly population is not necessary and not desirable (Schnabel 2016).

This paper focuses on the possibility of implementing this law in a morally sound way. Conceptually, euthanasia for a completed life is not sufficiently different to the form of euthanasia that is already legally and morally accepted in the Netherlands (Raus and Sterckx 2015). The rationale supporting the Dutch policy on euthanasia pertains to the principles of non-malevolence and autonomy (Beauchamp and Childress 2013; Rachels 1975; Seay 2011). The primary function of euthanasia in the Netherlands is to prevent unnecessary suffering, when the individual experiencing the suffering no longer wishes to continue and there is no prospect of the suffering ending by any other way than death (Overheid 2001; Rietjens et al., 2009; Beauchamp 2006; Schramme 2015). It can be convincingly argued that this is also the case in euthanasia for a completed life; the individual is experiencing an existential suffering and this has made the quality of life not worth living (Raus and Sterckx 2015; Pans 2006; Cholbi and Varelius 2015). At their essence, these two forms of euthanasia have the same ethical underpinning; an individual is suffering to the extent that their quality of life is critically diminished and they no longer view their life as one worth living. It is for this reason that the ethical permissibility on a purely conceptual level of allowing euthanasia based on a completed life is not what will be discussed in this article. As euthanasia based on medically founded suffering is already deemed sufficiently permissible to be allowed under certain conditions, it is taken as a starting premise that this should also be the case for those who experience their lives as complete, due to the fact both forms of euthanasia have similar ethical justifications, as previously stated. The significant points of difference between the two forms of euthanasia are in the implementation. Within this question of implementation, the two elements this article will expand on is firstly, the significance of certain eligibility criteria as stipulated in the law proposal, and secondly the complexities involved in the role of the “end-of-life coordinator,” a new position to be introduced specifically for this form of euthanasia.

I will discuss whether Pia Dijkstra’s vision for the implementation of euthanasia on the basis of a completed life is morally permissible. I focus on two ethically problematic elements of Dijkstra’s proposal: the dilemma of an age-dependent threshold and the nature of the end-of-life coordinator. Finally, I will conclude that although, in principle, euthanasia on the basis of a completed life could be morally permissible, it may be difficult to develop an implementation that is ethically acceptable. This is demonstrated by the fact that besides Pia Dijkstra’s vision, there is another, mutually exclusive perspective on how to implement euthanasia for a completed life, written by her party member Paul Schnabel. Different as these views are, they are both also highly problematic, suggesting that the formulation of an appropriate and morally acceptable implementation plan could be very difficult.

## The Dilemma of An Age Threshold

The question at the core of this dilemma is who is the desired target population for this law proposal, or what condition should make someone eligible for euthanasia under this law? A major point of contention within the debate on euthanasia for a completed life is whether or not to implement an age-specific threshold of eligibility. As it stands, the law proposal Pia Dijkstra wrote specifies that the requester must be over the age of seventy-five to qualify for euthanasia on the basis of a completed life (Wet Toetsing Levenseindebegeleiding van Ouderen op Verzoek 2016). This element of the law proposal is controversial and often used as an argument to oppose the passing of the proposal (NOS 2017). It is argued that an age threshold will always be arbitrary and that aiming this practice exclusively at elderly people could be discriminatory (Van Steenbergen, 2017b).

This question has given rise to a divide within the political party the author of the law proposal belongs to.^1^ Dijkstra explicitly envisions the law to be specifically for the elderly population (NOS 2017). Paul Schnabel, First Chamber member representing the same party, and his advice committee disagree. For Schnabel, the age of the individual requesting euthanasia is less important than the feelings they are experiencing. The advice committee states they reject the idea of an age threshold, not only because it is arbitrary but also not relevant, as everyone ages at their own pace (Schnabel 2016). I will consider each of these positions in turn; initially I will elaborate on Dijkstra’s views as set out in the proposal documentation, following which I will offer my own discussion and position concerning these arguments, and finally I will outline Schnabel’s position.

### Dijkstra’s Position

Dijkstra argues in her explanatory memorandum that the decision to implement an age-dependent threshold was made for three reasons. Firstly, people of a more mature age will be better able to estimate their future life perspective than those who are younger. The term “life perspective” refers to an individual’s prospect of the remaining years of their life and its holding a sufficiently good quality to make life preferable to death. Individuals over the age of seventy-five are reaching the end of their life, which means they have a clearer image of what is to come than a young person would. As Dijkstra puts it, there is less time for “unexpected twists and turns.” By this she means either a sudden change in life perspective or the sudden occurrence of events which could change one’s life perspective. The limited scope for this at an older age makes the estimation of whether one’s life remains worth living more accurate, according to Dijkstra.

Secondly, Dijkstra argues that the events that would typically cause the usual characteristics of a completed life are more likely to happen at an older age. As one grows older, the likelihood increases that one will grow lonely as the spouse dies or grow more physically dependent.

Finally, Dijkstra argues that the existence of an age threshold will be a source of peace, even for those who have not yet reached it. The knowledge that the age of eligibility is close could have a reassuring effect and paradoxically offer a sense of purpose or something to live towards.

### Response to Dijkstra’s Position

In response to Dijkstra’s first point, one could argue that according to the Dutch life expectancy, seventy-five-year-olds still have on average five years of life (Volksgezondheid en Zorg 2016). In practice it will usually be more than that, due to the principle that the closer one becomes to the average life expectancy, the more likely they are to become significantly older than the average (de Lange 2007a). Five years (at least) still constitute a large amount of time in which, as Dijkstra puts it, twists and turns are still entirely possible (Oeppen and Vaupel 2002; van der Heide et al. 2014). One could argue that a change in life perspective could happen at any time, regardless of how long one has left to live (Oeppen and Vaupel 2002). Moreover, this decreased likelihood for twists and turns is an empirical claim for which she offers no support. In fact, there is some empirical research to suggest that individuals experience a transformation in life perspective as they grow older and reach the end of their life (Braam et al. 1998; Kendig and Browning 2016; Boone James and Wink 2006). This research is by no means conclusive, but it does suggest that there is a significant uncertainty in estimating the likelihood of twists and turns at certain points in an individual’s life. However, Dijkstra’s argument does treat this likelihood with an undue amount of certainty. Furthermore, another factor which is at least as significant in establishing a person’s life perspective is the persistence of the death wish. Dijkstra ignores this element, while it is an essential factor in ascertaining the authenticity and sustainability of the request. If a sixty-year-old individual has been experiencing a continuous death wish for thirty years, it is highly unlikely they will have a sudden change in perspective. However, if a seventy-six-year-old has had a death wish for eight months, due to her husband’s recent death, there is a distinct possibility she will start to feel better as the initial grief starts to pass. It may be more helpful to evaluate how long this death wish has been present, rather than how long many years of life the requester has left (Schnabel 2016). What could be argued is that the physical and psychological symptoms which can potentially cause the experience of a completed life, such as dependence, loneliness, etc. will only increase (Gawande 2015). This is a valid point but slightly different to what Dijkstra is claiming here.

Regarding the second argument, this is certainly a valid point. Feelings such as loneliness, loss of identity and autonomy, loss of societal relevance are more likely to occur in old age. Specifically questions of loss of identity and societal relevance are specific to growing old (van Wijngaarden 2016; van Wijngaarden, Leget, and Goossensen 2015; de Lange 2007b; Cassell 2004). However, while I agree with Dijkstra’s observations concerning the likelihood of these events occurring in old age, this argument does not necessarily support the necessity of introducing an age threshold. This only means that those seeking this form of euthanasia will most likely be individuals of a certain age. This could even be interpreted as an argument for why an age threshold is not necessary. When left to take its natural course, the option of euthanasia for a completed life will largely attract people of a certain age, rendering a special policy to ensure this somewhat redundant.

Dijkstra’s third argument seems speculative and hypothetical. There is no current data on how elderly people still under the age threshold will feel about the threshold, as something like this has never occurred before and no empirical research has been done. However, for the sake of argument, let us assume that this point is true, and the prospect of becoming old enough to make such a request would offer these individuals a certain sense of peace. This sentiment is still not necessarily specific to an age threshold. One could say the same thing about the mere existence of the option to request suicide. Just the knowledge that one can make a choice to end their life in a safe and dignified way whenever they choose could offer sufficient peace to continue (Drion 1991). An age threshold is not necessary for this sense of peace.

A point Dijkstra does not address in her explanatory memorandum is the risk of stigma an age threshold poses. By making this option available for specifically the older generation, it may imply they are disposable. Allowing one specific population to decide when they want to die, implies that those who are not offered this option have importance outside their own narrative, importance an older generation is exempt from. This could enhance the feeling of societal irrelevance older people already widely suffer from, as van Wijngaarden has demonstrated (PBL 2018).

Furthermore, this feeling of societal irrelevance could transform from a personal insecurity to a more general attitude. It has been argued that an undesirable representation of age can be damaging, not only for the older population itself but also for how the population is seen by other demographic groups (Bytheway 2011). By implementing a policy that publicly implies irrelevance and even indifference towards a certain group, may cause a stigma towards elderly people. A stigma of this kind would mean that the feeling of aimlessness and pointlessness that older people already largely experience about themselves (van Wijngaarden 2016), could be shared by other populations in regard to older people. This could potentially make an already vulnerable population, even more so (Ash 2014). Such a development may cause the right to die to become a duty to die, possibly before the individual is truly ready to.

### Schnabel’s Position

As previously mentioned, Paul Schnabel and his advice committee are not in favour of an age threshold. Their view on how a completed should be constituted is more focussed on the completed life itself; the lack of a desirable life perspective and the persistence of the death wish as a result of that lack of perspective. As Dijkstra does, Schnabel acknowledges that this will refer mainly to elderly people but certainly not always. Schnabel argues that everyone ages at their own pace and that different people will reach a certain level of maturity at different ages. A completed life is not only subjective is its manifestation but also in the age at which it is reached. Schnabel’s definition of a completed life is also more general than the way it is usually described, as suffering is not an intrinsic element in Schnabel’s view. In most other definitions, a completed life is seen as a form of hopeless and unbearable suffering. For Schnabel this is not necessarily the case; there certainly could be suffering, but there could also be a mere general sense of contentment at having accomplished all existential goals and a feeling of needlessness to go on (Schnabel 2016).

Although this view seems to circumvent the issues posed by Dijkstra’s position, as this approach does not single out a specific population and thereby avoids the risk of the development of a stigma, it still has its own problems. Schnabel and his committee take a very general approach, both concerning the definition of a completed life and the hypothetical requester. One could argue this approach makes the process much more vague and undefined compared to how Pia Dijkstra views it. Not only can anyone apply for this euthanasia, establishing whether death is in their best interest becomes much more complex. Taking this general approach makes the possibility of implementing this expansion to the euthanasia law safely much more challenging, as every element is extremely vague and hard to verify. If individuals of all ages are able to apply for this form of euthanasia, the assessment of how sustainable and authentic the request is may be much more challenging and complex. Dijkstra’s argument is that the principle of a completed life is already very vague and to counter the risk attached to that interpretive freedom, there should be as much demarcation as possible.^2^

Additionally, waiting until the existing euthanasia law includes this type of euthanasia means keeping it within the medical domain, which opposes a crucial element of Dijkstra’s proposal. Keeping this practice in the medical domain invites the ethical problems surrounding the proper role of the physician (Kuhse and Singer 2009). The role of the physician in the context of traditional euthanasia is already highly contested (Pellegrino 2001). Specifically, this contention is with regard to the physician’s duties of beneficence and non-maleficence. Euthanasia has historically divided opinion on whether this is the physician performing the ultimate harm or the ultimate relief of suffering (Gaylin et al. 1995; Andersson et al. 2010; Bradley 2017; Zaidi 2014; Bosshard et al. 2008; KNMG 2011; Misbin 1995). This divide will only be further enhanced when, as Schnabel considers entirely possible and valid, there is no suffering at all but just a sense of overall contentment and a reluctance to continue living. It could very easily be argued that a physician’s duty is to heal those who are suffering, and while this can mean performing euthanasia on a patient who cannot be offered relief in any other way, this duty does not extend to individuals who are not ill or suffering in any way (Gaylin et al. 1995; Garcia 2007).

In conclusion, while these two views both argue for the option to die whenever one chooses, they take two entirely different stances on the practical implementation of this option. Dijkstra views her proposal as a way of lifting this specific form of euthanasia from the medical realm and allowing it specifically for older people in attempt to counter the risk attached to the interpretive freedom inherent in the subjectivity of the experience of a completed life. Schnabel takes a more general approach, both regarding the concept of a completed life and the type of person that should be allowed to request this form of euthanasia. He argues for a natural expansion of the existing euthanasia law, rather than a separate and new law.

Both these approaches are problematic in different ways. These problems are not impossible to solve but both Dijkstra and Schnabel must offer more explanation on how they plan to do so. At present, neither position is fit to implement. Both could become workable and morally acceptable, but for this to be possible, additional information and explanation is still required. For Dijkstra this means offering a response to the risk of an ageist stigma developing, and for Schnabel the problems lie in the increased uncertainty of the sustainability and authenticity of the request and the difficulties surrounding the role of the physician.

## The Nature of the End-of-Life Coordinator

The end-of-life coordinator, her responsibilities and ethical issues is an element of the debate that has remained entirely unaddressed in the public discussion. Given that this area has been discussed and researched so little, my contribution is exploratory. My aim is not to offer a definite answer to this problem but rather to open up the discussion and hopefully start the discourse. Introducing this as a new profession has of course not been discussed in the general euthanasia debate as it is specific to this form of euthanasia but even in the focussed debate on completed life nothing has been written about this role and its ethical implications. Due to the fact that so little has been written about this element, this section will rely on my own analysis of the official documentation, more than the rest of this article.

As previously mentioned, the law proposal aims to remove this practice from the medical domain.^3^ The rationale is that the foundation of the feelings the requester is experiencing need not necessarily be medical (Wet Toetsting Levenseindebegeleiding op Verzoek 2016). The strictly medical physician will be expected to address existential questions to a certain extent; it is after all expected that the physician empathize sufficiently with the patient to determine whether their suffering is unbearable (Overheid 2001). However, this ability may not always sufficiently extend to every case of a fulfilled life. A problem here is that although the foundation of the problem may not be medical, medical training will still be necessary for the end-of-life coordinator to perform the euthanasia safely and comfortably. It is for this reason that Dijkstra is suggesting to create an entirely new vocation, specifically dedicated to this form of euthanasia. The whole procedure will be performed by an end-of-life coordinator. The end-of-life coordinator will be medically trained (in order to fulfil the requirement that the procedure be carried out in a medically careful and safe way) but with additional training, comparable to how a teaching training would be complementary to a primary degree. During this specialized training, the prospective coordinator will learn how to appropriately relate to existential questions concerning detachment, loss of identity, and loss of purpose (Memorie van Toelichting 2016).

The explanatory memorandum states the end-of-life coordinator has three main responsibilities:

1. Establish that the requester came to the decision of requesting euthanasia without external pressure,
2. Establish that the decision is well-considered and durable, and
3. Prescribe the lethal medication to the requester, and remain present when the medication is taken.

### The Practical and Ethical Dimensions of Assessment and Decision-Making

It is the task of the end-of-life coordinator to discover whether the request was free from pressure and whether the request is well-considered and sustainable. To do this, the law proposal stipulates that there must be at least two extensive and incisive consultation sessions over the course of at least two months (Wet Toetsting Levenseindebegeleiding op Verzoek 2016). During these two meetings, the coordinator must gain the conviction that the request is authentic and sustainable and that the suffering of the requester is indeed unbearable. These duties are exactly the same as those of the physician under the current legislation. The coordinator must also discuss alternative options with the requester, although the requester has the right to decline all such offers. The coordinator must also inform the requester of all procedural aspects. After this is complete, the requester must submit their final decision in writing or through audio-visual means. Following this, the coordinator must consult a second, independent coordinator, who must meet with the requester at least once. If this secondary coordinator’s opinion does not interfere with the initial coordinator’s conclusion, the initial coordinator will arrange a date and time with the requester (Memorie van Toelichting 2016).

Estimating whether the request is well-considered and sustainable is a difficult and complex task. One could question whether it is reasonable to make the performance of this intricate judgement with such drastic and irreversible consequences an intrinsic role in a profession.

This expectation is to a certain extent inextricably linked to euthanasia as a practice and more generally to the medical field. Physicians must make these judgements all the time (Raus and Sterckx 2015). Not only for traditional euthanasia is this same question relevant but also in matters of discontinuing life supporting treatment. Doctors must often make decisions about who will benefit the most from scarce resources (Savulescu 2015; Bullock 2015; Miller, Truog and Brock 2009). This would suggest that in itself, it is morally permissible to include decision-making regarding the respective benefits of life and death a role within a profession. A significant difference, however, is that the form of euthanasia currently accepted in the Dutch legislation circumvents this difficulty to a certain extent. Although there is still uncertainty in the sense it is impossible to know for a fact how much another person is suffering (Schopenhauer 2014), a physician is still able to relatively accurately estimate the curability and prognosis for a certain disease. He will be able to inform a patient of their prospects. Related to point, a physician has both the medical prognosis of the disease and an empathetic assessment to rely on in order to make a decision. However, research suggests that physicians rely on the physical elements of the disease and on curability much more heavily than they do on the empathy-based judgement regarding the unbearableness of the suffering, resulting in GPs granting euthanasia requests much less when the illness has no physical (and thereby more conveniently quantifiable) manifestations (van Tol, Rietjens, and van der Heide 2010). This is a significant difference between the role of the physician and that of the end-of-life coordinator. The latter has been entirely stripped of that concretely quantifiable measuring tool and must only rely on empathy. Although the physician must also rely on empathy to a certain extent, they evidently avoid doing so and base themselves more on the curability of the disease (van Tol, Rietjens, and van der Heide 2010; Florijn 2018). However, in the completed life context, there is no disease and thereby no prognosis. The end-of-life coordinator must not only factor in the uncertainty that comes with estimating the suffering of someone else but additionally there is the uncertainty of the persistence of the cause of the suffering.

Another important difference between the role this sort of decision-making plays for a physician and for an end-of-life coordinator is the following. For a physician, as previously mentioned, the core responsibility is to heal. There is of course some interpretive discord on what that realistically entails (Quill and Byock 2000), but that does not take away from the consensus that, in principle, this is the primary duty. In this capacity, making decisions on whether or not to end a life (either for the relief of suffering or for justice reasons), will amount to only a relatively small part of the physician’s professional activities. Additionally, due to the more scientific and medically oriented context, the doctor will have a clearer prognosis and will be able to better inform their patient about the possible outcomes of their situation. They are able to weigh up relatively accurately whether treatment will help, whether there are other options for relieving the suffering of the patient, or whether the scarce resource is much better spent on another patient (Buiting et al. 2011).

Deciding on whether or not to euthanize is the end-of-life coordinator’s only job. Where the primary duty of the doctor is to heal, the primary duty of the end-of-life coordinator is to decide whether euthanasia is appropriate for the requester. Although they are able to refer them to other healthcare professionals, there is no indication that they will be able to perform this alternative healing themselves. Furthermore, this decision which constitutes a core part of their profession will be inevitably based on inherently vague criteria. As discussed in the section on the concept of a completed life, it is widely agreed that a solid definition is not achievable, due to the highly subjective nature of the experience (de Lange 2015).

In itself, there is nothing wrong with expecting one to make difficult end-of-life decisions as a part of one’s profession (Selzer 1995). These decisions will always need to be made, and therefore it is unreasonable and detrimental to attempt to deem them morally objectionable in the professional context (Fins and Bacchetta 1995; Hook 1995). However, when these difficult decisions become the focal point of a profession, its primary duty, it becomes more problematic. One could argue that when a person’s job is entirely dedicated to making these decisions, their expertise and level of experience only makes them increasingly able to do the job well. This may be the case when the judgement criteria are not so fundamentally vague. The process of deciding whether the decision of the requester is sustainable and authentic is so uncertain and open to interpretation, that the profession will most likely come with a significant sense of doubt; not only about decisions currently at hand but about past decisions. This doubt could over time become burdensome. It could be questioned whether this is too much pressure for someone to be able to viably cope with on a daily basis as their job.

### The Duty of the End-of-Life Coordinator to Be Present During the Procedure

The continuous pressure discussed above also plays a role in the moral questionability of the end-of-life coordinator having to be present for the euthanasia itself. They must be there as the requester ingests the lethal medication to confirm she is taking it correctly, the medication is going to the right person, and to step in if something goes wrong. This measure is taken to ensure that the procedure is conducted safely and correctly (Memorie van Toelichting 2016). However, this obligation is arguably problematic in other ways.

A key requirement for the end-of-life coordinator to be able to do their job well and accurately, is the ability to be empathetic (Vink 2011). In the short time they have to spend with the requester they must establish highly personal and intricate details about the requester. They must gain the conviction that death is indeed in the best interest of this individual, and due to the lack of a medical foundation, this must be done purely through conversation with the requester. If the request is successful, the end-of-life coordinator must then be present during the death of this individual whom they have been forced to get to know on a very deep and personal level, albeit in a very short time. The individual in question may wish to have their family and friends present or they may choose not to. In any scenario, the death of this individual will usually be a highly emotionally charged situation. To perform this procedure on a regular basis as a part of one’s job could potentially become extremely demanding. It puts an excessive amount of emotional strain on the individual performing the euthanasia. It could be argued that the end-of-life coordinator may be reassured by the feeling they are ultimately helping or relieving suffering. This is valid, but this is also the case for a physician in the context of traditional, medical euthanasia, but even so it has been shown that performing euthanasia has a severe, emotionally detrimental effect on physicians (Stevens 2006; van Marwijk et al. 2007). Of course, it is not unusual for people to be emotionally affected in the context of performing a stressful or emotionally taxing job; the same could be said of physicians who must often deal with their patients dying (Mitchell 2004) or researchers who must perform painful experiments on animals. However, the fundamental difference here is that none of those other emotionally trying jobs require empathy as a core requirement to do the job well. It is certainly helpful if doctors are empathetic towards their patients, but it does not disproportionately influence their ability to cure disease if they are not. Researchers are conforming to a scientific method and their prime objective is to obtain results on whatever it is they are researching (Mukerjee 1997). Again, empathy is not a central part of their profession. The two crucial requirements to be an end-of-life coordinator, however, is to be sufficiently empathetic to engage with the existential suffering of another person and to be sufficiently resilient to withstand the very regular experience of death that they themselves brought about. These two qualities are sufficiently at odds with each other to make them very hard to reconcile within one person and one profession. It is possible that this seeming dissonance is addressed in the training one undergoes to become an end-of-life coordinator, but Dijkstra does not address this in her law proposal or her explanatory memorandum.

In conclusion, introducing the end-of-life coordinator as a new and separate profession poses two important problems. The first is the potential burden caused by the regular decision whether or not to euthanize based on something very subjective and vague. This decision is arguably more clear-cut for a physician in a traditional euthanasia context, as there is more clarity on the prognosis of the disease and the patient’s prospects. The end-of-life coordinator must make their decision based exclusively on their conversations with the requester. Secondly, it could be argued that the duty to stay present throughout the entire euthanasia process may give rise to a conflict of qualities. To come to a decision regarding the request, the end-of-life coordinator must possess a certain level of empathy. However, simultaneously, the coordinator must also possess sufficient resilience and a certain stoicism to be able to regularly experience the emotionally loaded situation of a person’s death. Although these are not insurmountable problems, they are problems that require a proposed solution. So far, Dijkstra has not addressed these potential issues, and in fact, no one has. These issues brought about by the concept of an end-of-life coordinator have been entirely left behind in the public debate, while they are significant problems. Until Dijkstra offers a retort to these risks, the proposal is not suitable for implementation.

## Conclusion

The debate on euthanasia for a completed life has been ongoing for over two decades, but it was Pia Dijkstra’s law proposal in 2016 which made the debate more immediate. The proposal is currently in the research stage and therefore highly topical and societally relevant in The Netherlands. For this discussion, I have taken as a starting premise that euthanasia for a completed life should in principle be acceptable.

The two points of discussion, the dilemma of an age threshold and the nature of the end-of-life coordinator have demonstrated persistent problems. These two issues indicate a divide within the D66 party between Dijkstra and Schnabel.

These two positions are each highly problematic in very different ways. For Dijkstra the problems pertain to the risk of creating a societal ageist stigma, and the difficulty surrounding introducing “end-of-life coordinator” as a new profession. For Schnabel, there is the increased possibility of misuse of the policy and the ethical issues surrounding the duty of the physician regarding individuals who are not ill. These problems form a recurring theme throughout this article, along with the fact that neither Dijkstra or Schnabel has offered a potential solution to these problems.

In the current state of affairs, this unfortunately means that neither position is realistic or fit for implementation. This is not to say that neither of these plans can ever be executed. Dijkstra’s proposal could be acceptable at a future time, but first she must offer more explanation regarding the problems posed by her proposal and how she plans to minimize the problems I have outlined in this article. However, the radical extent to which the two positions differ, and how problematic they both still are, suggests that finding a morally justifiable method of implementation for this (in theory ethically acceptable) concept may be very difficult.

## Footnotes

1 Daniël Boomsma, in conversation.

2 Daniël Boomsma, in conversation.

3 Daniël Boomsma, in conversation.
